# Trends in Cigarette Smoking Among United States Adolescents

**DOI:** 10.31486/toj.23.0113

**Published:** 2023

**Authors:** Maria C. Mejia, Adedamola Adele, Robert S. Levine, Charles H. Hennekens, Panagiota Kitsantas

**Affiliations:** ^1^ Department of Family and Community Medicine, Baylor College of Medicine, Houston, TX; ^2^ Department of Biomedical Science, Charles E. Schmidt College of Medicine, Florida Atlantic University, Boca Raton, FL; ^3^ Department of Family Medicine, Charles E. Schmidt College of Medicine, Florida Atlantic University, Boca Raton, FL; ^4^ Charles E. Schmidt College of Medicine, Florida Atlantic University, Boca Raton, FL; ^5^ Department of Population Health and Social Medicine, Charles E. Schmidt College of Medicine, Florida Atlantic University, Boca Raton, FL

**Keywords:** *Adolescent*, *cigarette smoking*, *smoking*

## Abstract

**Background:** Cigarette smoking remains the leading avoidable cause of premature death in the United States, accounting for approximately 500,000, or 1 in 5, deaths annually. We explored trends in cigarette smoking among US adolescents.

**Methods:** We used data for adolescents in grades 9 through 12 from 1991 to 2021 from the Youth Risk Behavior Survey provided by the US Centers for Disease Control and Prevention. We explored trends overall as well as by sex, race/ethnicity, and school grade.

**Results:** All cigarette use—assessed as ever, occasional, frequent, or daily—among adolescents declined markedly from 1991 to 2021. Specifically, ever use significantly decreased from 70.1% in 1991 to 17.8% in 2021 (*P*<0.05), an almost 4-fold decline. Occasional use significantly decreased from 27.5% in 1991 to 3.8% in 2021 (*P*<0.05), a greater than 7-fold decline. Frequent use significantly decreased from 12.7% to 0.7%, a greater than 18-fold decline. Daily use declined from 9.8% in 1991 to 0.6% in 2021, a greater than 16-fold decline. Cigarette smoking significantly decreased from 1999 to 2021 across sex, race/ethnicity, and school grade (*P*<0.05). In 2021, daily use was higher in boys vs girls; Hispanic/Latino and White youth vs Black and Asian youth; and 12th graders vs 9th, 10th, and 11th graders.

**Conclusion:** These data show large and significant decreases in cigarette use among US adolescents in high school grades 9 through 12 from 1991 to 2021. Nonetheless, the data also suggest residual clinical and public health challenges that will require targeted interventions.

## INTRODUCTION

In the United States, almost 500,000 premature deaths every year are attributable to cigarette use. Thus, cigarette smoking causes about 1 in 5 US deaths each year and remains the leading single avoidable cause of premature death.^1,2^

Cigarette smoking is associated with cardiovascular disease and cancer. For prevention of smoking-related cardiovascular disease, benefits begin to accrue within weeks of cessation and reach the risk level of the nonsmoker within 3 to 5 years, even among the elderly. In contrast, for cancer prevention, smoking cessation benefits do not even begin to accrue for 3 to 5 years. Thus, for cardiovascular disease, it is never too late to quit, but for cancer, it is never too early.^3^

Although smoking rates have declined significantly since 1965, early onset of tobacco use causes nicotine dependence and less willingness and ability to quit.^2,4,5^ Efforts to prevent and treat early-onset cigarette use during adolescence could have major clinical and public health impacts.^6^

For this original research report, we explored overall trends in cigarette use among a large sample of US adolescents in high school grades 9 through 12 from 1991 to 2021, as well as by sex, race/ethnicity, and each school grade. Considering the serious health-related risks associated with smoking and the variations that exist in smoking behaviors in youth by demographic characteristics,^7^ examining patterns of use over time within this population provides important insights.

## METHODS

### Data and Analytic Sample

Data were obtained from the 1991 to 2021 Youth Risk Behavior Survey, a cross-sectional, school-based survey that is publicly available from the Centers for Disease Control and Prevention. The survey monitors health-related behaviors that contribute to the leading causes of morbidity and mortality, and data are collected every 2 years from students in school grades 9 through 12 in the 50 US states and the District of Columbia. Mpofu et al provide details about the Youth Risk Behavior Survey sampling, data collection methods, response rates, and other methodologic details.^8^

The study sample consisted of 226,076 US high school students attending private or public schools and in grades 9 through 12. The sample included data from 1991 (n=12,115), 1993 (n=16,155), 1995 (n=10,473), 1997 (n=15,124), 1999 (n=14,888), 2001 (n=13,238), 2003 (n=14,739), 2005 (n=13,284), 2007 (n=13,601), 2009 (n=15,888), 2011 (n=14,743), 2013 (n=13,203), 2015 (n=14,989), 2017 (n=14,407), 2019 (n=12,317), and 2021 (n=16,912). The sample size for each year reflects the largest number of responses, taking into consideration all of the smoking behaviors included in this study; in some cases, the number of responses for an individual behavior in a given year is lower than these annual numbers. Missing response data averaged approximately 2% in the annual surveys. Findings are reported in accordance with Strengthening of the Reporting of Observational Studies in Epidemiology (STROBE) guidelines.^9^ This original research was considered exempt by the Institutional Review Board of the Baylor College of Medicine.

### Measures

The cigarette smoking measures included (1) ever tried cigarette smoking, which was defined as taking even 1 or 2 puffs; (2) currently smoked cigarettes occasionally, which was defined as smoking on at least 1 day during the 30 days before the survey; (3) currently smoked cigarettes frequently, which was defined as smoking on 20 or more days during the 30 days before the survey; and (4) currently smoked cigarettes daily, which was defined as smoking on all 30 days during the 30 days before the survey.^10^ We assessed these parameters of cigarette use overall, as well as by sex (boy/girl), race/ethnicity (Black, Asian, Hispanic/Latino, and White), and school grade (9th, 10th, 11th, and 12th).

### Data Analysis

We used percent changes in the use of cigarettes among the sample from 1991 to 2021 as our measure of effect and 95% CIs to test for statistical significance. Statistically significant differences in cigarette smoking were considered at levels of *P*<0.05.

## RESULTS

### Overall Changes in Cigarette Use

Cigarette use declined markedly from 1991 to 2021 in all usage categories: ever, occasional, frequent, and daily (Table 1). Specifically, ever use significantly decreased from 70.1% in 1991 to 17.8% in 2021 (*P*<0.05), an almost 4-fold decline. Occasional use significantly decreased from 27.5% in 1991 to 3.8% in 2021 (*P*<0.05), a greater than 7-fold decline. Frequent use significantly decreased from 12.7% to 0.7% (*P*<0.05), a greater than 18-fold decline. Daily use declined from 9.8% in 1991 to 0.6% in 2021, a greater than 16-fold decline.

**Table 1.** Cigarette Use Among United States Adolescents, 1991 vs 2021

	Cigarette Use by Year, % (95% CI)^a^	
Cigarette Usage Category	1991	2021
Ever	70.1 (67.8-72.3)	17.8 (15.9-19.9)
Occasional	27.5 (24.8-30.3)	3.8 (3.3-4.4)
Frequent	12.7 (10.6-15.3)	0.7 (0.6-1.0)
Daily	9.8 (7.8-12.2)	0.6 (0.4-0.8)

^a^Statistically significant differences (*P*<0.05) were observed in cigarette use between 1991 and 2021 across all categories of cigarette use.

Note: Ever is defined as taking even 1 or 2 puffs; occasional is defined as smoking on at least 1 day during the 30 days before completing the Youth Risk Behavior Survey; frequent is defined as smoking on 20 or more days during the 30 days before the survey; and daily is defined as smoking on all 30 days during the 30 days before the survey.

### Cigarette Use by Sex

Cigarette smoking in all usage categories (ever, occasional, frequent, and daily) significantly decreased from 1999 to 2021 in both boys and girls (Table 2).

**Table 2.** Cigarette Use Among United States Adolescents by Sex, 1991 vs 2021

		Cigarette Use by Year, % (95% CI)^a^	
Cigarette Usage Category	Sex	1991	2021
Ever	Boys	70.6 (68.6-72.6)	17.8 (16.0-19.8)
	Girls	69.5 (66.4-72.5)	17.7 (15.2-20.4)
	Overall	70.1 (67.8-72.3)	17.8 (15.9-19.9)
Occasional	Boys	27.6 (24.6-30.9)	3.9 (3.1-4.9)
	Girls	27.3 (23.9-31.0)	3.7 (3.2-4.4)
	Overall	27.5 (24.8-30.3)	3.8 (3.3-4.4)
Frequent	Boys	13.0 (11.2-15.1)	0.9 (0.7-1.4)
	Girls	12.4 (9.8-15.7)	0.4 (0.3-0.8)
	Overall	12.7 (10.6-15.3)	0.7 (0.6-1.0)
Daily	Boys	10.5 (8.6-12.6)	0.8 (0.6-1.0)
	Girls	9.1 (6.8-11.9)	0.3 (0.2-0.6)
	Overall	9.8 (7.8-12.2)	0.6 (0.4-0.8)

^a^Statistically significant differences (*P*<0.05) were observed in cigarette use between 1991 and 2021 for boys and girls.

Note: Ever is defined as taking even 1 or 2 puffs; occasional is defined as smoking on at least 1 day during the 30 days before completing the Youth Risk Behavior Survey; frequent is defined as smoking on 20 or more days during the 30 days before the survey; and daily is defined as smoking on all 30 days during the 30 days before the survey.

**Figure 1.**
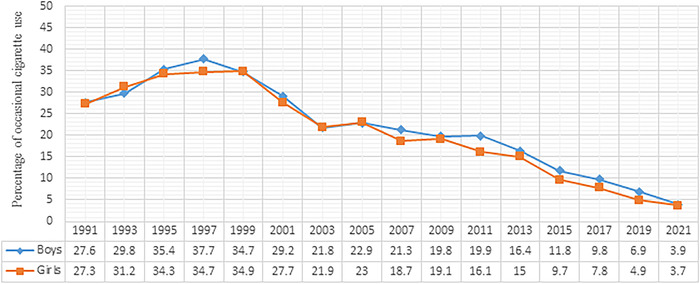
Trend of occasional cigarette use among United States adolescents by sex, 1991 to 2021.

The percentage of high school boys who ever tried cigarettes declined significantly from 70.6% in 1991 to 17.8% in 2021 (*P*<0.05), an almost 4-fold decline. Frequent cigarette use also decreased significantly in boys from 13.0% in 1991 to 0.9% in 2021 (*P*<0.05), a greater than 14-fold decline. Daily cigarette use among boys significantly decreased from 10.5% in 1991 to 0.8% in 2021 (*P*<0.05), a greater than 13-fold decrease. Occasional cigarette use in boys was 27.6% in 1991, rose initially to its highest level of 37.7% in 1997, and then declined to 3.9% by 2021 (Figure 1).

In girls, ever cigarette use declined significantly from 69.5% in 1991 to 17.7% in 2021 (*P*<0.05), an almost 4-fold decline. Frequent cigarette use in girls significantly decreased from 12.4% in 1991 to 0.4% in 2021 (*P*<0.05), a 31-fold decrease. Daily cigarette use among girls also significantly decreased from 9.1% in 1991 to 0.3% in 2021 (*P*<0.05), a 30-fold decline. In girls, occasional cigarette use was 27.3% in 1991, rose initially to its highest level of 34.9% in 1999, and then declined to 3.7% by 2021 (Figure 1).

### Cigarette Use by Race/Ethnicity

Overall, steep declines in cigarette use during the past 3 decades were observed across all racial/ethnic groups. However, by 2021, the 3.1% of Hispanic/Latino and 4.8% of White youths who reported occasional smoking remained significantly higher (*P*<0.05) than the percentage of occasional smokers in the other racial/ethnic groups (Table 3).

**Table 3.** Cigarette Use Among United States Adolescents by Race/Ethnicity, 1991 vs 2021

		Cigarette Use by Year, % (95% CI)^a^	
Cigarette Usage Category	Race/Ethnicity	1991	2021
Ever	Black	67.2 (63.8-70.5)	11.5 (8.9-14.6)
	Asian	55.4 (49.9-60.9)	7.1 (4.2-11.7)
	Hispanic/Latino	75.3 (73.6-76.9)	19.4 (16.6-22.5)
	White	70.4 (67.6-73.1)	19.4 (17.6-21.4)
	Overall	70.1 (67.8-72.3)	17.8 (15.9-19.9)
Occasional	Black	12.6 (10.2-15.5)	1.7 (1.1-2.8)
	Asian	14.6 (10.5-20.0)	0.6 (0.2-1.9)
	Hispanic/Latino	25.3 (22.5-28.2)	3.1 (2.4-3.9)
	White	30.9 (27.6-34.5)	4.8 (4.0-5.7)
	Overall	27.5 (24.8-30.3)	3.8 (3.3-4.4)
Frequent	Black	3.1 (2.2-4.5)	0.5 (0.2-1.2)
	Asian	6.6 (4.0-10.6)	0.3 (0.1-1.3)
	Hispanic/Latino	6.8 (5.4-8.5)	0.8 (0.5-1.2)
	White	15.4 (12.8-18.5)	0.8 (0.5-1.2)
	Overall	12.7 (10.6-15.3)	0.7 (0.6-1.0)
Daily	Black	2.5 (1.7-3.7)	0.5 (0.2-1.2)
	Asian	4.2 (1.9-9.1)	0.3 (0.1-1.3)
	Hispanic/Latino	4.0 (3.1-5.1)	0.6 (0.3-1.2)
	White	12.2 (9.6-15.3)	0.6 (0.4-0.9)
	Overall	9.8 (7.8-12.2)	0.6 (0.4-0.8)

^a^Statistically significant differences (*P*<0.05) were observed in cigarette use between 1991 and 2021 across all racial/ethnic groups.

Note: Ever is defined as taking even 1 or 2 puffs; occasional is defined as smoking on at least 1 day during the 30 days before completing the Youth Risk Behavior Survey; frequent is defined as smoking on 20 or more days during the 30 days before the survey; and daily is defined as smoking on all 30 days during the 30 days before the survey.

**Figure 2.**
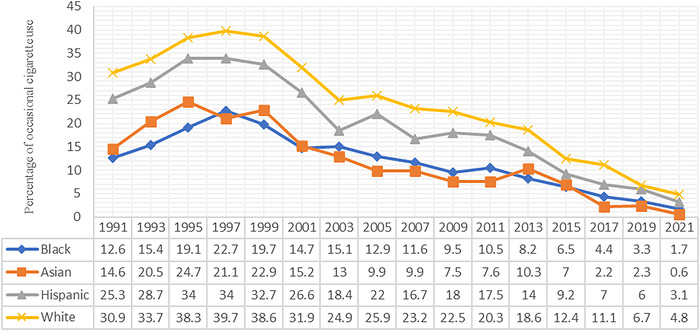
Trend of occasional cigarette use among United States adolescents by race/ethnicity, 1991 to 2021.

In Black youth, ever cigarette use declined significantly from 67.2% in 1991 to 11.5% in 2021 (*P*<0.05), an almost 6-fold decrease. In Black youth, frequent cigarette use significantly decreased from 3.1% in 1991 to 0.5% in 2021, while daily use decreased from 2.5% in 1991 to 0.5% in 2021. Occasional cigarette smoking among Black youth increased from 12.6% in 1991 to 22.7% in 1997 and then decreased to 1.7% in 2021, although slight increases occurred in 2003 and 2011 (Figure 2).

In Asian youth, ever cigarette use declined significantly from 55.4% in 1991 to 7.1% in 2021 (*P*<0.05), an almost 8-fold decrease. Frequent cigarette use decreased from 6.6% in 1991 to 0.3% in 2021. Similarly, daily use among Asian youth decreased from 4.2% in 1991 to 0.3% in 2021. Occasional smoking among Asian youth increased from 14.6% in 1991 to 24.7% in 1995, decreased to 21.1% in 1997, and despite small increases in 1999 and 2013, declined to 0.6% in 2021 (Figure 2).

In Hispanic/Latino youth, ever cigarette use declined significantly from 75.3% in 1991 to 19.4% in 2021 (*P*<0.05), an almost 4-fold decrease (Table 3). Occasional use among Hispanic/Latino youth increased from 1991 to 1997 (25.3% vs 34%, respectively) and then steadily decreased over time (Figure 2).

In White youth, ever cigarette use declined significantly from 70.4% in 1991 to 19.4% in 2021 (*P*<0.05), a more than 3-fold decrease. Frequent cigarette use among White youth decreased from 15.4% in 1991 to 0.8% in 2021, and daily use declined from 12.2% in 1991 to 0.6% in 2021. Occasional use was 30.9% in 1991, the percentage increased until 1997, and then it steadily declined to 4.8% in 2021 (Figure 2).

### Cigarette Use by High School Grade

**Figure 3.**
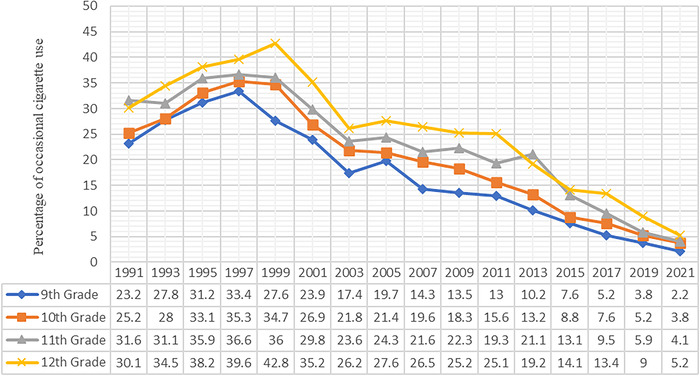
Trend of occasional cigarette use among United States adolescents by high school grade, 1991 to 2021.

Overall, a significant decline in cigarette use was observed in 2021 relative to 1991 across all high school grades (Table 4). Occasional cigarette use increased from 1991 to 1997 among students in high school grades 9, 10, and 11 and then declined thereafter (Figure 3). For students in high school grade 12, the increase in occasional cigarette use continued through 1999 and then decreased.

**Table 4.** Cigarette Use Among United States Adolescents by High School Grade, 1991 vs 2021

		Cigarette Use by Year, % (95% CI)^a^	
Cigarette Usage Category	High School Grade	1991	2021
Ever	9	64.8 (61.6-67.9)	13.9 (11.6-16.6)
	10	68.3 (64.8-71.6)	16.4 (13.9-19.2)
	11	72.8 (69.3-76.0)	18.3 (16.1-20.7)
	12	74.5 (71.2-77.6)	22.7 (19.7-26.0)
	Overall	70.1 (67.8-72.3)	17.8 (15.9-19.9)
Occasional	9	23.2 (19.5-27.4)	2.2 (1.6-3.0)
	10	25.2 (22.5-28.1)	3.8 (3.0-4.8)
	11	31.6 (27.8-35.7)	4.1 (3.4-5.0)
	12	30.1 (25.7-34.8)	5.2 (4.4-6.2)
	Overall	27.5 (24.8-30.3)	3.8 (3.3-4.4)
Frequent	9	8.4 (6.2-11.3)	0.4 (0.2-0.8)
	10	11.3 (9.0-14.1)	0.6 (0.3-1.3)
	11	15.6 (12.9-18.8)	0.8 (0.4-1.4)
	12	15.6 (12.4-19.4)	1.0 (0.7-1.5)
	Overall	12.7 (10.6-15.3)	0.7 (0.6-1.0)
Daily	9	6.4 (4.6-8.7)	0.3 (0.1-0.7)
	10	8.8 (6.5-11.8)	0.4 (0.2-0.9)
	11	12.2 (9.7-15.2)	0.6 (0.3-1.0)
	12	12.2 (9.7-15.2)	0.9 (0.6-1.3)
	Overall	9.8 (7.8-12.2)	0.6 (0.4-0.8)

^a^Statistically significant differences (*P*<0.05) were observed in cigarette use between 1991 and 2021 across all high school grades.

Note: Ever is defined as taking even 1 or 2 puffs; occasional is defined as smoking on at least 1 day during the 30 days before completing the Youth Risk Behavior Survey; frequent is defined as smoking on 20 or more days during the 30 days before the survey; and daily is defined as smoking on all 30 days during the 30 days before the survey.

In 2021, US adolescents in 12th grade had a significantly higher percentage of occasional smokers at 5.2% compared to students in all other high school grades (*P*<0.05): 4.1% for 11th grade, 3.8% for 10th grade, and 2.2% for 9th grade.

In 9th graders (14 to 15 years old), ever cigarette use declined significantly from 64.8% in 1991 to 13.9% in 2021 (*P*<0.05), an almost 5-fold decline. Frequent cigarette use decreased from 8.4% in 1991 to 0.4% in 2021, and daily use decreased from 6.4% in 1991 to 0.3% in 2021.

In 10th graders (15 to 16 years old), ever cigarette use declined significantly from 68.3% in 1991 to 16.4% in 2021 (*P*<0.05), a more than 4-fold decline. Frequent cigarette use decreased from 11.3% in 1991 to 0.6% in 2021, and daily use decreased from 8.8% in 1991 to 0.4% in 2021.

In 11th graders (16 to 17 years old), ever cigarette use declined significantly from 72.8% in 1991 to 18.3% in 2021 (*P*<0.05), an almost 4-fold decline. Frequent cigarette use decreased from 15.6% in 1991 to 0.8% in 2021, and daily use decreased from 12.2% in 1991 to 0.6% in 2021.

In 12th graders (17 to 18 years old), ever cigarette use declined significantly from 74.5% in 1991 to 22.7% in 2021 (*P*<0.05), a more than 3-fold decline. Frequent cigarette use decreased from 15.6% in 1991 to 1.0% in 2021, and daily use decreased from 12.2% in 1991 to 0.9% in 2021.

## DISCUSSION

These data show major and significant declines in cigarette use spanning 3 decades, from 1991 to 2021, among a large sample of US adolescents. Ever use decreased almost 4-fold, the occasional use decline was greater than 7-fold, and the decline in frequent use was greater than 18-fold. Daily use declined from 9.8% in 1991 to 0.6% in 2021, a greater than 16-fold decline. Smoking rates were almost equal in boys and girls across the 4 categories of cigarette usage examined. Overall, inequalities in cigarette use among adolescents have been present for decades by both sex and race/ethnicity. However, by 2021, discrepancies in smoking cigarettes by sex were diminished. With respect to race/ethnicity, by 2021, the decreases in cigarette use were even more pronounced among Black and Asian adolescents, while the rates among Hispanic/Latino and White adolescents remained higher than the smoking rates among Black and Asian adolescents but were still significantly lower than the 1997 rates.

Interestingly, while all grades experienced a significant decline in cigarette use, 12th graders consistently reported the highest percentage of occasional smokers compared to the other school grades, even in 2021. This finding suggests that while smoking has decreased across all age groups, older adolescents might still be more prone to experimenting with cigarettes than their younger counterparts.

These reassuring decreasing trends in traditional cigarette use stand in stark contrast to the rise in the popularity of e-cigarettes among adolescents.^11^

These cross-sectional data are descriptive and useful to formulate but not to test hypotheses.^12^ Nonetheless, they contribute to the formulation of hypotheses that are consistent with other studies demonstrating successful strategies and programs that have been implemented. For example, public health campaigns such as the Centers for Disease Control and Prevention Tips From Former Smokers and the Truth Initiative have been targeting youths with antismoking messages, making them aware of the dangers of cigarette smoking.^13,14^ Policy changes such as the implementation of higher tobacco taxes, the enforcement of smoke-free laws, and the legal age increase for tobacco purchases might have deterred younger individuals from accessing and using tobacco products.^15^ In addition, school-based interventions including programs such as Tobacco-Free Generations have been launched in many schools, which not only educate students about the risks of smoking but also engage them in anti-tobacco advocacy.^16^

Behavioral interventions targeting smoking in youths have consistently shown short-term benefits. Although some behavioral interventions might show immediate effects, implementing and maintaining these effects over the long term can be challenging.^17^ Although effective programs may be available, ensuring that all adolescents have equal access to these interventions, especially in rural or underserved areas, is a significant challenge. Interventions might need adjustments based on cultural, socioeconomic, or geographic differences. A one-size-fits-all approach may not be effective for diverse youth populations.

Peer smoking is a strong predictor of adolescent smoking initiation and continuation. Behavioral interventions must address the challenge of peer influence, which can often counteract cessation efforts.^18^ For example, according to 2021 observations from the Kentucky Incentives for Prevention, peer interventions were more effective than individual interventions among adolescents in producing increased awareness of the hazards of cigarette smoking and greater reported intentions to quit.^19^

Speculating about the observed declines in cigarette smoking that continued during the coronavirus disease 2019 pandemic across sex, race/ethnicity, and school grade is tempting. Plausibly, increased awareness of preserving respiratory health and the reduced social and peer pressure that resulted from spending more time at home may have contributed to these declines. These hypotheses could be tested in analytic studies.

Several limitations are associated with the Youth Risk Behavior Survey, as the survey relies on self-reported data that can be subject to biases. Students may underreport or exaggerate their smoking behaviors because of recall bias, social desirability bias, or fear of repercussion. The survey also collects data only from students attending school. Adolescents who have dropped out or are absent on the day of the survey might be at higher risk for smoking and other risky behaviors. Finally, with the rise in popularity of e-cigarettes and vaping devices, traditional cigarette use might decline or change. The strengths of these data are the large sample size and the assessment of patterns in cigarette smoking across 3 decades.

## CONCLUSION

The substantial decrease in cigarette use among US adolescents from 1991 to 2021 is an encouraging public health achievement. This decrease underscores the importance of continued vigilance, research, and intervention to further reduce tobacco use and its associated harms. While these results show reassuring trends in cigarette use in US adolescents during the last 30 years, they also suggest residual clinical and public health challenges that will require targeted interventions. Future directions for research include exploring the transition from cigarette use to e-cigarettes/vaping among adolescents and understanding the associated initiating factors and health impacts. Conducting longitudinal studies that track the smoking behaviors of adolescents into adulthood and exploring life-course impacts and patterns could generate important information for tobacco prevention and control strategies.

## Notes

*This article meets the Accreditation Council for Graduate Medical Education and the American Board of Medical Specialties Maintenance of Certification competencies for Patient Care, Medical Knowledge, and Systems-Based Practice.*
